# Potential mechanism of phytochemical-induced apoptosis in human prostate adenocarcinoma cells: Therapeutic synergy in genistein and β-lapachone combination treatment

**DOI:** 10.1186/1475-2867-4-5

**Published:** 2004-08-17

**Authors:** James Kumi-Diaka, Simone Saddler-Shawnette, Alex Aller, Jayann Brown

**Affiliations:** 1 Department of Biological Sciences, Schmidt College of Science, Florida Atlantic University @ Davie, 2912, College Avenue, Davie FL. 33314, USA; 2 Rambaugh-Goodwin Cancer Research Institute, Sunrise, FL. USA

## Abstract

**Background:**

Prostate cancer is the second leading cause of male death in the United States. The incidence increases most rapidly with age, and multiple genetic and epigenetic factors have been implicated in the initiation, progression, and metastasis of the cancer. Nevertheless, scientific knowledge of the molecular mechanisms underlying the disease is still limited; and hence treatment has only been partially successful. The objective of the current studies was to examine the role of caspase 3 (CPP32) and NAD(P)H:quinone oxidoreductase (NQO1) in the signaling of genistein-and β-lapachone (bLap)-induced apoptosis in human prostate carcinoma cells PC3.

**Results:**

Both genistein and bLap produced dose-dependent growth inhibition and treatment-induced apoptosis in PC3. Treatment with caspase 3 inhibitor, DEVD-fmk before exposure to genistein, significantly inhibited caspase 3 expression and treatment-induced apoptosis; implicating CPP32 as the main target in genistein-induced apoptosis in PC3. Contrary to this observation, inhibition of CPP32 did not significantly influence bLap-induced apoptosis; implying that the major target of bLap-induced apoptosis may not be the caspase. Treatment with NQO1 inhibitor, dicoumarol (50 μM), prior to exposure of PC3 to bLap led to significant decrease in bLap toxicity concurrent with significant decrease in treatment-induced apoptosis; thus implicating NQO1 as the major target in β-lapachone-induced apoptosis in PC3. In addition, the data demonstrated that NQO1 is the major target in bLap-genistein (combination)-induced apoptosis. On the contrary, blocking NQO1 activity did not significantly affect genistein-induced apoptosis; implying that NQO1 pathway may not be the main target for genistein-induced apoptosis in PC3 cells. Furthermore, blocking NQO1 and CPP32 did not confer 100% protection against genistein-induced or bLap-induced apoptosis.

**Conclusion:**

The data thus demonstrate that both genistein-and bLap-induced apoptosis are mostly but not completely dependent on CPP32 and NQO1 respectively. Other minor alternate death pathways may be involved. This suggests that some death receptor signals do not utilize the caspase CPP32 and/or the NQO1 death pathways in PC3. The demonstrated synergism between genistein and bLap justifies consideration of these phytochemicals in chemotherapeutic strategic planning.

## Background

Prostate cancer is the most common non-skin malignancy and the second leading cause of male death in the United States [1]. The incidence of prostate cancer increases most rapidly with age, and multiple genetic and epigenetic factors have been implicated in the initiation, progression, and metastasis of prostate cancer. Nevertheless, scientific knowledge of the molecular mechanisms underlying the disease is still limited.

The problem often faced with the clinical management of prostate cancer is derived not only from the fact that no single gene or molecule can serve as a reliable marker [2,3], but also that there is still no effective therapeutic regimen available without serious, sometimes fatal side effects. Unfortunately, at the time of clinical diagnosis, human prostate cancers mostly present themselves as heterogeneous entities – hormone-dependent and hormone-independent, and proliferating and non-proliferating. The tumor re-growth that occurs after post-treatment remission is largely due to progression of initially androgen-dependent to androgen-independent cancer cell [4] and/or non-proliferating to proliferating tumor cells. Therefore chemotherapeutic strategies should focus on eradicating all cancer cells irrespective of state of growth or sensitivity to hormone. This calls for a search for drug combination treatment that works through different mechanism of action. The facts that prostate cancer cells retain the inherent apoptotic machinery potentially subject them to an appropriate efficacious chemotherapeutic intervention.

The molecular mechanism(s) and intracellular mediators of both spontaneous-and treatment-induced apoptosis are not fully elucidated. However, evidence from several research investigations seem to indicate that a variety of stimuli, including physiological, pathologic, environmental or cytotoxic, can trigger the process of apoptosis in many mammalian cells [5,6], and that both apoptosis and necrosis may share some upstream events in the molecular pathways that lead to induction of apoptosis [7-11]. An emerging strategy for cancer chemotherapy is the choice of drugs that induce apoptosis and/or disruption of angiogenesis with eventual elimination of the cancer. It is suggested that blocking of caspase activation in an apoptotic process may divert apoptotic cell death to a necrotic demise [10]; implying that apoptosis and necrosis may share some upstream events in the molecular pathways of apoptosis induction. Among the dietary phytochemicals of potential therapeutic significance, are genistein isoflavone and β-lapachone, both of which induce apoptosis and also inhibit angiogenesis (genistein) in an array of cancer cells [6,12,13].

Genistein isoflavone [4',5',]-trihydroxyisoflavone) is a metabolite of soy [14] and has a heterocyclic, diphenolic structure similar to estrogen [14]. The phytochemical isoflavonoid family to which genistein belongs is a group of plant chemicals that resemble steroid estrogens and mimic their biological reactions [15,6,16]. Several clinical studies indicate that genistein has some chemoprotective and chemotherapeutic potential against many tumors, including prostate, breast, and colon cancers through several mechanisms of action including: apoptosis induction; modulation of cell cycle activity by arresting cell cycle at the G_2_-M stage [17]; inhibition of DNA topoisomerase-II and tyrosine protein kinase [18]; competitive inhibitor of ATP binding to the catalytic domain of tyrosine kinase [14,18]; stimulating the production of sex hormone-binding globulin (SHBG), which may lower the risk of hormone related cancers by decreasing the amount of free and active hormones in the blood [19,20].

The other phytochemical of potential therapeutic significance is β-lapachone [3, 4-dihydro-2, 2-dimethyl-2H-naphtol (1,2-b) pyran-5,6-dione], a simple plant product with a chemical structure different from currently used anti-cancer drugs. It has been previously demonstrated that the primary mode of cytotoxicity of β-Lapachone is through the induction of apoptosis [21,22]. Structural similarities between β-lapachone and other members of the naphthoquinone family, such as menadione, suggest that the enzyme, NAD(P)H:Quinone oxidoreductase enzyme (NQO1) may be involved in the activation or detoxification of β-lapachone [23-25].

While a number of *in vitro *effects of β-lapachone and genistein have been described, knowledge of the key intracellular targets of β-lapachone and genistein is limited. Recent reports have suggested that by β-lapachone-induced apoptosis is non-caspase mediated in breast [26] and prostate cancer cells [21,34], and that the cytotocxicity of this compound is dependent on the activity of NAD(P)H:Quinone oxidoreductase enzyme (NQO1/xip3) [26,27]. B-Lapachone has been shown to be an inhibitor of DNA repair that sensitizes cells to DNA-damaging agents [28,29]. It directly inhibits DNA topoisomerase I and II [30-32] and induces a cell-cycle delay in G_1 _and/or S phase followed by apoptotic and/or necrotic cell death [13]. The apoptosis induced by β-lapachone is p53 independent [21], and has been associated with upregulation of Bak as well as cleavage of caspase-7 [33] and caspase 3 in a variety of mammalian cells [13,33].

The objective of this study was to determine the potential chemosensitivity of human prostate adenocarcinoma, PC3 cells to β-lapachone and Genistein and the role of caspase 3 (CPP32) and NAD(P)H:Quinone oxidoreductase enzyme (NQO1) in the signaling of β-Lapachone and genistein-induced apoptosis in PC3 cells. The hypothesis is that combination treatment with the two phytochemicals will be strongly preventive and/or interceptive against prostate cancer by modulating epigenetic events (apoptosis) associated with the progression of active and latent cancer cells to clinical malignancy.

## Results

### Genistein and β-lapachone inhibit growth and proliferation of human prostate carcinoma cells, PC3

**Figure 1:**
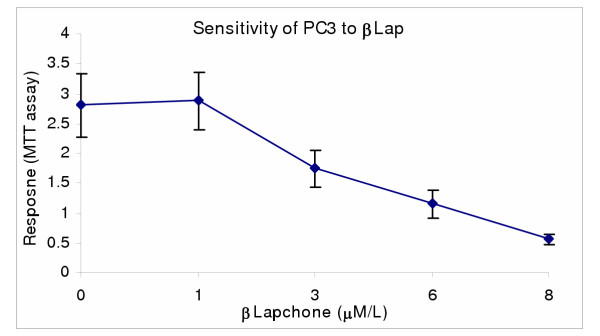
β-lapachone-induced growth inhibition in PC3. PC3 cells (1 × 10^4 ^cells/well) were cultured in 24-well plates for 48 hr to allow 85–90% confluence; treated with varyingconcentrations of bLap and assessed for post-treatment viabilitywith the MTS assay. Note the dose-dependent growth inhibitionin PC3. Data points represent means ± SEM of three independentexperiments performed in triplicates

**Figure 2:**
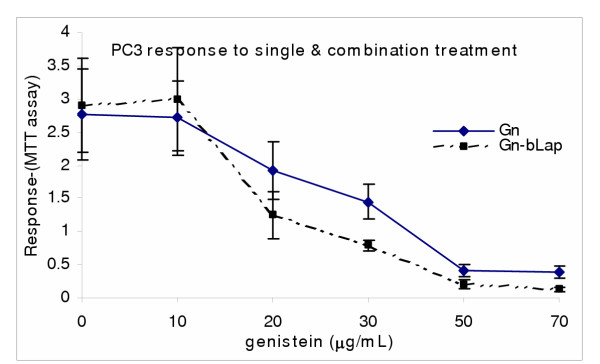
Genistein (Gn)/β-Lapachone combination treatment of PC3. Cells were treated as described in the methods and subjected to post-treatment viability with MTS colorimetric assay. Data points represent the means ± SEM of three independent experiments performed in triplicates.

**Figure 3:**
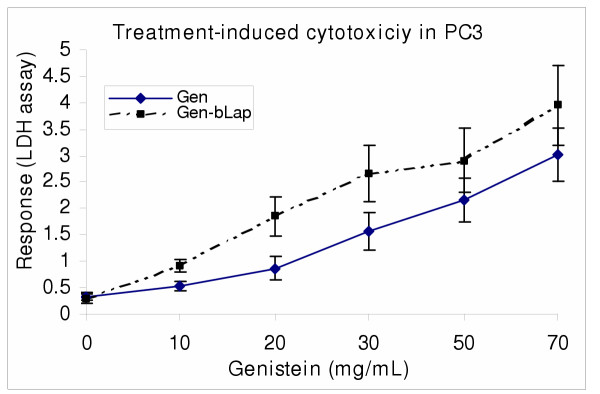
Single and combination of PC3 cells with genistein (Gn) and β-lapachone (bLap) βLap. Briefly, PC3 cells were seeded at 1 × 10^4 ^cells/well in 48-well MTP and co-cultured with Gn_0-70 _with/without bLap (1.2 μM); followed by determination of treatment-induced cytotoxicity as described in the methods. Data points represent means ± SEM of three experiments performed in triplicates

Human prostate carcinoma cells PC3, was used to determine the chemosensitivity of prostate cancer to genistein isoflavone and β-lapachone *in vitro *using Trypan blue exclusion, LDH and MTS bioassays. In single and combination treatments, both genistein and β-lapachone inhibited cell growth and decreased cell survival through induction of cell death [Figures. 1,2,3]. The data indicated that PC3 sensitivity to both single and combination treatment is dose-dependent, and that PC3 was significantly more sensitive (P < 0.05) to the combination treatment than to the single treatment; indicating a potential synergism between genistein and β-lapachone [Figures 2,3].

### Genistein and β-lapachone induce apoptosis in human prostate cancer cells

**Figure 4:**
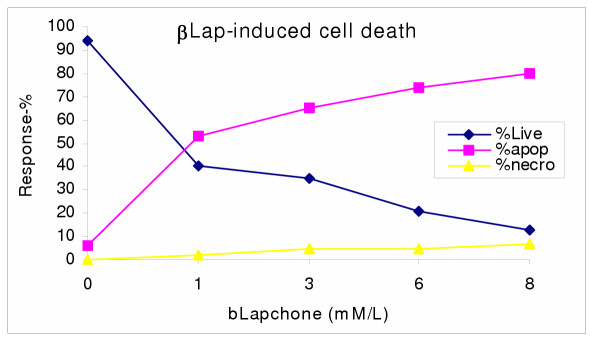
βLap)-induced cell death in PC3 cells. PC3 cells were co-cultured with varying concentrations of bLap; and and the degree of treatment-induced apoptosis and/or necrosis assessed with the Annexin V-FITC assay, as described in the methods. Data points are means ± SEM of three independent experiments performed in triplicates.

**Figure 5:**
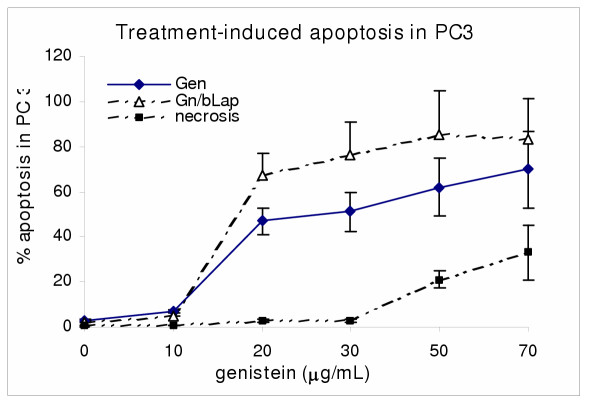
Combination treatment-induced cell death in PC3 cells. PC3 cells were co-cultured with varying concentrations of Gn (Gn_0-70_) with or without bLap (1.2 μM), and apoptotic/necrotic cell death assessed with theAnnexin V-FITC assay as described in the methods. Data points are means ± SEM of three independent experiments performed in triplicates

Extensive cell death was observed in proliferating human prostate cancer cells after treatment with β-lapachone and genistein isoflavone. To determine if the treatment-induced cell occurred through cytotoxic necrosis and/or apoptosis, cells were harvested and assayed for apoptosis induction with Annexin V-FITC and TUNEL apoptosis assays to detect early and late apoptosis respectively. Aliquots of cells were also stained with acridine orange/ethidium bromide nuclear stain to distinguish between apoptotic and necrotic cells. The results revealed that in both single and combination treatments, cell death was mostly through apoptosis in a dose-dependent manner [Figures 4,5]. With increasing concentration of the agents, cell death through necrosis increased correspondingly. Furthermore, combination treatment induced significantly more apoptosis in PC3 (p <0.01) than individual treatment with either agent.

### Dicoumarol enhanced the survival of human prostate cancer cells (PC3) following single treatments with β-lapachone (bLap) and Gn/bLap combination but not in PC3 cells treated with genistein alone

**Figure 6:**
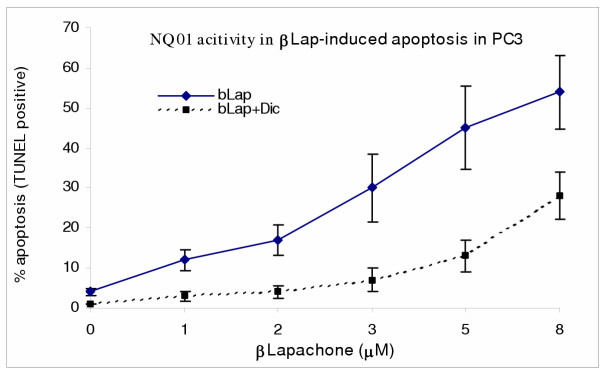
Role of NQO 1 in β-Lapaphone-mediated apoptosis in PC3 cells. PC3 cells were treated with bLap alone or in combination with 50 μM dicoumarol (NQO 1 inhibitor) as described in the methods; and TUNEL assays performed to monitor apoptosis. Data points represent means + SEM of three independent experiments performed in triplicates.

**Figure 7:**
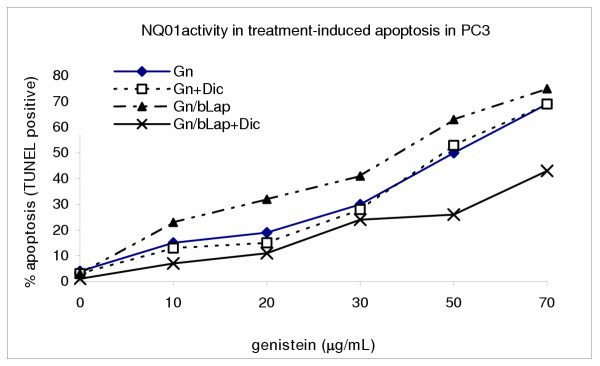
NQO1 is the main target in bLap/Gn-induced apoptosis in PC3 cells. Cells were treated with genistein (Gn) and Gn/bLap combination with or without 50 μM dicoumarol as described in the methods; and TUNEL assays performed to monitor apoptosis. Data points represent means ± SEM of three independent experiments performed in triplicates.

To determine the potential role of the enzyme NAD(P)H:quinone oxidoreductase (NQO1) in β-lapachone (bLap)-and genistein (Gn)-induced apoptosis in PC3, the cells were exposed to Gn and bLap in the presence or absence of dicoumarol in single and combination treatments; and then assayed for apoptosis by the Annexin V-FITC and TUNEL assays. Dicoumarol is a specific inhibitor of NQO1. The results revealed that blocking NQO1 activity with dicoumarol (50 μM) significantly reduced bLap-induced apoptosis [Figure 6]; indicating that bLap-induced apoptosis requires involvement of NQO1 target. However, dicoumarol did not appear to have significant effect on Gn-induced apoptosis [Fig 7]; indicating that NQO1 did not play significant role in Gn-induced apoptosis. [Figures 6,7]. The degree of apoptosis induction was highest in the Gn-bLap combination treatment without inhibiting NQO1 activity with dicoumarol [Figure 7]; implying that a possible synergy between Gn and bLap may be due to NQO1 activity.

### Activation of CPP32 in genistein-induced apoptosis in PC3 but not in β-lapachone-induced apoptosis in PC3

**Figure 8:**
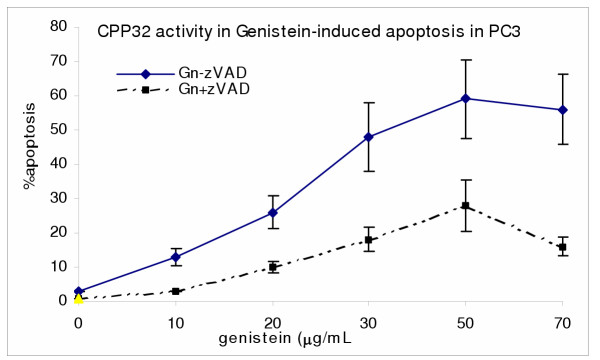
Caspase-3 (CPP32) activity in genistein-induced apoptosis in PC3 cells. PC3 cells (2.5 × 10^3 ^cells/well) were cultured; then treated with/without 100 μM caspase inhibitor (zVAD-fmk) for 2 hr; and then with 10–70 μg/mL genistein for 4 hr as described in the methods. Cells were thenanalyzed for caspase (CPP32) activity and corresponding apoptosis in the cells. Data points were the means ± SEM of two independent experiments performed in triplicates.

**Figure 9:**
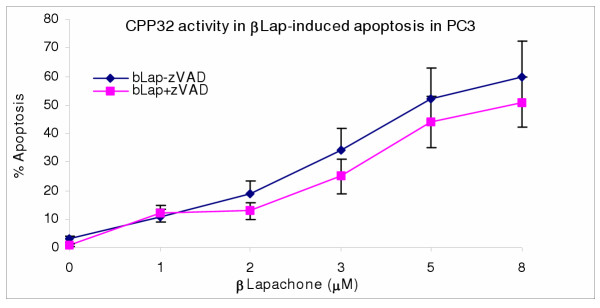
CPP32 is the major pathway in genistein-induced apoptosis in PC3 cells. PC3 cells (2.5 × 10^3 ^cells/well) were cultured in 48-well culture plates; treated with/without 100 μM caspase inhibitor (zVAD-fmk) for 2 hr; then with 1–8 μM β-Lapachone (bLap) for 4 hr as described in the methods. Cells were then analyzed for caspase (CPP32) activity and corresponding apoptosis. Data pointsare the means ± SEM of two independent experiments performed in triplicates

To determine if apoptosis induced by β-lapachone and/or genistein involved activation of caspase 3 protease (CPP32), the PC3 cells, were subjected to treatments with Gn and/or bLap co-administered with or without CPP32 inhibitor (DEVD-fmk), and then cultured as previously described. Post-treatment apoptosis was determined as previously described. As shown in Figures 8 and 9, blocking the release of caspase 3 significantly decreased genistein induced apoptosis but not bLap-induced apoptosis; indicating the significant role of CPP32 in the molecular pathway of Gn-induced apoptosis; and minor involvement of CPP32 in bLap-induced apoptosis in PC3. Furthermore, blocking CPP32 activity did not significantly affect combination treatment-induced apoptosis (Figure not shown).

## Discussion and Conclusions

In this study, we determined the role of caspase 3 (CPP32) and the enzyme NAD(P)H:quinone oxidoreductase (NQO1) in the signaling of β-lapachone (bLap)-and genistein (Gn)-induced apoptosis in human prostate adenocarcinoma, PC3 cells. Data from this study demonstrate significant inhibition of cell growth and proliferation in PC3 cells, with significant difference in chemosensitivity of PC3 to genistein and β-lapachone (P < 0.01). Furthermore, growth inhibition of PC3 cells strongly correlated with the MTS and LDH assay results. The pattern of response and percent post-treatment live cells was consistent with previous results [6,37-39]. The genistein-and bLap-induced morphological changes observed in the cells were identical in pattern but differed in severity at a given exposure time; indicative of differences in chemosensitivity of PC3 to genistein and β-lapachone. These observations were consistent with previous results [5,6]. Furthermore, previous studies have shown that β-lap [22] induces morphologic changes indicative of apoptosis in human breast cancer cells. Similar alterations in morphology including cell shrinkage and chromatin condensation in the PC3 cells following single and combination treatment with β-lapachone and genistein isoflavone.

The present data also implicates caspase-3 protease, CPP32, in the molecular pathway of genistein-induced apoptosis in prostate PC3 cancer cells, consistent with previous investigations [10,11,39]. Using the caspase inhibitor DEVD-fmk, caspase activity was arrested concurrent with significant decrease in genistein-induced apoptosis in PC3 cells. However, it is noteworthy that inhibition of caspase did not confer 100% protection against genistein-induced apoptosis; implying alternative death pathways, which suggests that some death receptor signals do not utilize the caspase CPP32 death pathways in PC3. We have previously demonstrated the significant role of caspase-3 protease in the genistein-induced apoptosis pathway in both testes and prostate (PC3) cancer cells [38,39].

The present data indicate a possible alternate CPP32 pathway in bLap-induced apoptosis in PC3. However, unlike the observation in genistein-induced apoptosis, blocking the CPP32 activity with the specific caspase inhibitor, DEVD-fmk, did not significantly change the percentage of bLap-induced apoptosis in PC3 cells; indicating that CPP32 many not be the main death pathway of bLap-induced apoptosis in PC3 cells. Activation of the caspase 3 in bLap-induced apoptosis has been reported in previous studies [10].

The potential role of NAD(P)H:quinone oxydoreductase (NQO1) activity in genistein-and bLap-induced apoptosis in PC3 was investigated. Co-culture of PC3 cells with dicoumarol, a specific inhibitor of NQO1 activity, significantly reduced the cytotoxicity of β-Lapachone in PC3 cells, as reflected in the significant reduction in the percentage of treatment-induced apoptosis. Dicoumarol increased cell survival. These results implicate NQO1 as the main target in bLap-induced apoptosis in PC3, consistent with previous observations [26,34,40]. However, the fact that blocking of NQO1 did not confer 100% protection against induction of apoptosis indicates a possible alternate pathway in bLap-induced apoptosis. The present data indicate some involvement of caspase protease CPP32, though not with the same significance as NQO1. The activation of cysteine protease has been observed after bLap treatment [26]. Pink et al [26] reported activation of the cysteine protease in MCF-7 and T4D breast cancer cells in bLap-induced apoptosis.

Contrary to the observation in bLap-induced apoptosis, blocking NQO1 activity did not significantly influence genistein-induced apoptosis in PC3 cells; implying NQO1 may not be the major target in genistein-induced apoptosis in PC3 cells. However, the overall data indicate a synergistic effect of genistein-bLap combination treatment of PC3 and, that the major target in the combination treatment-induced apoptosis in PC3 cells is NQO1. Investigation into genistein/bLap synergism in a number human cancer cells is on-going.

### Conclusion

It is concluded from the data obtained that: i) both genistein and bLap exert growth inhibition effects in PC3 cells, with significant differences in chemosensitivity of PC3 to the two agents; ii) there is a manifestation of synergism between genistein-bLap combination treatment; iii) both genistein and bLap induce apoptosis in PC3 cells; iv) the major target in genistein-induced apoptosis in PC3 cells seems to be CPP32; v) the major molecular target in bLap-induced apoptosis in PC3 cells appear to be NQO1; vi) the major target in the genistein-bLap combination treatment-induced apoptosis appears to be NQO1; and vii) combination treatment appears significantly more efficacious than single treatments. More extensive studies are ongoing to delineate and clarify the molecular mechanisms underlying the combination effects.

## Materials and Methods

### Chemicals

Genistein isoflavone (Indoline Chemical Co. Summerville, NJ, USA) was constituted in DMSO (dimethylsulfoxide) solvent as 10, 20, 30, 50 and 70 μg/ml solutions (G_10-70_) and frozen at -37°C until used. β-lapachone (Sigma Scientific (St. Louis, MO, USA) was constituted in DMSO solvent as 1, 2, 3, 5 and 8 μM/ml solutions (bL_1-8_) and stored at -37°C. Dicoumarol (Sigma Scientific St. Louis, MO, USA) was constituted in DMSO as 50 μM/ml and frozen at -37°C until used. The caspase 3 inhibitor, DEVD-fmk and substrate DEVD-afc were purchased from Biovision (Palo Alto, CA). Culture media (RPMI 1640), antibiotics, trypsin-EDTA, and other reagents were purchased from Sigma scientific (St. Louis, MO, USA).

### Cell lines

Human prostate adenocarcinoma (PC3) was a generous donation from Rambaugh-Goodwin Cancer Research Institute, Plantation FL.

### Cell Culture

To assess the chemosensitivity of human prostate cancer cells to single and combination treatment with genistein (Gn) and β-lapachone (bLap), cells were sub-cultured under 5% CO_2 _at 37°C for 48 hrs to reach 80–90% confluence. All cells were grown and maintained as monolayers in 25 m^2 ^tissue culture flasks (Sigma Scientific, St. louis, MO, USA) in RPMI 1640 containing 15 mM HEPES, and supplemented with 0.45% glucose (w/w), 5.0% FBS and 100 U^. ^mL^-1 ^penicillin + 100 mg^. ^ml^-1 ^streptomycin. The cells were then harvested by gentle scraping with a cell scraper. The cell suspensions were then grown at a density of 2.5 × 10^3 ^cells/well in 24-well microtiter plates (MTP) for 36 hr to allow adherence. The supernatants were discarded and the agents (Gn or bLap) were added over a range of 5 cytotoxic concentrations in single and combination treatments. In preliminary studies with bL_1-8_, the IC_50 _ranged between 1.8–3 μM for a number of cells. Therefore in the present studies, 2.0 μM (bL_2_) was used in the combination studies with varying concentrations of genistein. All treatments were in triplicates. Dicoumarol was added to the cells and incubated for 4 hr before treatment with either genistein or β-lapachone alone or in combination. All plates were then incubated at 37°C in a humidified atmosphere of 5% CO_2 _in air for a maximum of 72 hr. At 12, 24 and 36 hr of incubation, 100 μl of the supernatant from each well was gently aspirated and replenished with 100 μl of fresh media. The supernatants were stored at -37°C until assayed for lactate dehydrogenase (LDH) enzyme activity. At 36 hr incubation, the cells were harvested by trypsinization with trypsin-EDTA, and processed for post-treatment metabolic activity using cell viability and apoptotic assays as described.

### A. Cell Viability Assays

#### A1.1 MTS assay

MTS assay depends on mitochondrial enzyme reduction of MTS solution to detect and determine cell viability. The MTS cell proliferation assay is a colorimetric method for determining the number of viable cells in proliferation. It is composed of solutions of a tetrazolium compound [3-(4,5-dimethylthiazol-2-yl)-5-(3-carboxymethoxyphenyl)-2-(4-sulfophenyl)-2H-tetrazolium, inner salt; MTS] and an electron coupling reagent (phenazine ethosulfate; PES). MTS is bioreduced by the cells into formazan product that is soluble in cell culture medium. Following cell culture as described above, 100 μL of cells were harvested from each treatment group and added to a 96 MTP followed by addition of 20 μl of MTS (2.5 mg/mL: Sigma Chemical Co) stock solution to each well. After 2 hr incubation under standard conditions of 5% CO_2 _and 37°C, the purple formazan product (indicative of reduction of MTS) was visible. The absorbance was read on Multiskan biochromatic automated microplate reader (Multiskan, DC) at 490 nm. The signal generated (color intensity) is directly proportional to the number of viable (metabolically active) cells in the wells. Relative cell numbers can therefore be determined based on the optical absorbance (optical density, OD) of the sample. The blank values were subtracted from each well of the treated cells and controls; and the mean and standard error for each treatment (singles and combination) were calculated relative to the control:



where A_C _= absorbance of the control (mean value): A_T _= absorbance of the treated cells (mean value)

A_B _= absorbance of the blank (mean value)

#### A1.2 Trypan Blue exclusion assay

For the Trypan blue exclusion test, cells were treated and cultured as described. They were harvested and Trypan blue dye solution was added to the cell suspensions. Total cell counts and viable cell number (survival rate) were determined by a standard hemocytometer procedure. Live-viable cells were seen as colorless (impermeable to the dye due to intact cell membrane) and dead cells were seen as blue (permeable to dye due to disruption of cell membrane):



### A2. LDH assay

Lactase dehydrogenase activity was measured by a non-radioactive protocol using the LDH cytotox kit (Cat. #. 1644 793: Boehringer-Mannheim GmbG, Bochemica). The previously frozen supernatants were thawed for LDH determination. Briefly, 100 μL/well of each cell-free supernatant was transferred in triplicate into wells in a 96-well microtiter plate (MTP) and 100 μL of LDH-assay reaction mixture (Kit: dye-catalyst mixture) added to each well. After 90 min incubation under standard conditions the absorbance of the color generated was read on Multiskan biochromatic automatic microplate reader at 490 nm. The mean absorbance/optical density (OD) for each treatment group was calculated. The blank values were subtracted from each well and the mean percent treatment-induced cytotoxicity for each cell line and treatment type (single and combination) was calculated as:



Where:

ABS_expt _= mean absorbance from the treated cells: ABS_low _= mean absorbance from controls (untreated cells)(spontaneous release of LDH)

ABS_hi _= mean absorbance from Triton X-100 treated cells (standard/maximum LDH release)(positive control).

### B. Detection of Treatment-induced Apoptosis

Treatment-induced apoptosis was assessed by two independent assays, Annexin V-FITC assay and the DNA Fragmentation (TUNEL) assay. PC3 cells were treated and co-cultured with the test agents as previously described in this study; and the subjected to the apoptosis determination assays as below:

### B1.1 Annexin V-FITC assay

Apoptosis-associated translocation of phosphatidylserine from the inner to the outer leaflet of the plasma membrane in GC27 and K833 cells was assessed with the use of FITC-labeled Annexin V, a calcium-dependent phospholipid-binding protein with a high affinity for phosphatidylserine; using AnnexinV-FITC Staining Kit (Boehringer Mannheim). Briefly, 100 μl aliquots of the previously prepared cell suspensions were centrifuged, and the cell pellets re-suspended in Annexin binding buffer, incubated with AnnexinV-FITC substrate; then cells were smeared onto microscope slides and either evaluated immediately with fluorescence microscope, or smears were fixed with 4% depolymerized paraformaldehyde and stored at -40°C for later examination as previously described [35]. Percentage of apoptosis in the cells was quantified based on morphological and fluorescence characteristics of apoptotic cells as previously described [5,35,36]. All tests were run in triplicates.

### B1.2 DNA Fragmentation (TUNEL) assay

The presence of apoptosis was determined by terminal deoxynucleotidyl transferase (TdT)-mediated dUTP nick end labeling (TUNEL), using the ApopTag^R ^kit (Boehringer Mannheim Co, Indianapolis, IN) as previously described [37]. The kit reagents detect apoptotic cells *in situ *by specific end labeling and detection of DNA fragments produced by the apoptotic process. To perform the TUNEL assay, slides of the PBS suspended cells were fixed with 4% paraformaldehyde for 30 minutes. The cells (slides) were then permeabilized with Triton X-100 at 4°C for 2 min; then flooded with TdT enzyme and digoxigenin-dUTP reaction buffer (TUNEL) reagent for 60 min in a humidity chamber at 37°C, washed with distilled water, incubated for 10 minutes with streptavidin-horseradish peroxidase complex. The stained mounted cells were examined at 100×, 200× and 400× magnification of the microscope (Olympus BH-2). Cell death was quantified by counting 150 cells in 5–7 separate fields of view per slide, and noting the percentage of apoptotic cells based on morphological appearance, as previously described [5,36].

### C Potential mechanism(s) of action

The potential involvement of caspase-3 protease (CPP32) and/or the enzyme NQO1 [NAD(P)H:quinone oxidoreductase] in the molecular pathways of β-lapachone-and/or genistein-induced growth inhibition and apoptosis in PC3 cells were determined, after treatment of the cells as already described.

### C1.1 Caspase-3 expression/activity in treatment-induced apoptosis

In order to determine the potential role of caspase-3 proteases (CPP32) in the common pathways of β-lapachone and genistein-induced growth inhibition and apoptosis, human prostate cancer cell lines were treated as previously described above. The activity of caspase 3 was determined using a the fluorometric substrate DEVD-afc and caspase 3 inhibitor DEVD-fmk according to the protocol of the Caspase Activity Assay kit.

Briefly, PC3 cells were treated and incubated as previously described. At 24, 48 and 72 hr cells were scrapped into suspension and centrifuged at 10,000 rpm for 10 min. The pellet was resuspended in 100 μl of lysis buffer and incubated at 4°C for 10 min, followed by centrifugation at 10,000 rpm for 10 min. Fifty μl aliquots of the supernatants were removed and placed in a 96-well microtiter plate (MTP) containing reaction buffer. The DEVD-afc substrate was added and the MTP was incubated at 37°C for 30 min. Activity was monitored with the linear cleavage and release of the afc side chain; and compared with a linear standard curve generated by the controls on the same MTP.

### C1.2 NQO1 Activity in treatment-induced apoptosis

In order to determine the potential role of enzyme NQO1 [NAD(P)H:quinone oxidoreductase] in the molecular pathways of β-lapachone-and genistein-induced growth inhibition and apoptosis in human prostate cancer, PC3 cell lines were treated as previously described. Dicoumarol (3-3'-methylene-bis (4-hydroxycou-marin) is a commonly used inhibitor of NQO1, which competes with NADH or NADPH for binding to the oxidized form of NQO1. Dicoumarol thereby prevents reduction and activation of various target quinines like β-lapachone. The cells, cultured as previously described, were treated concomitantly in single and combination treatments of varying concentrations β-lapachone (bLap), genistein (gen), and bLap-Gn combination with and without 50 μM dicoumarol as previously described. The treated cells were harvested and tested for treatment-induced apoptosis by the methods previously described in this study.

## Authors' contributions

JKD contributed 50% in all aspects of the research. All other authors contributed equally-50%

All authors read and approved the final manuscript.
